# Supplementary material for: MicroRNA-9 controls dendritic development by targeting REST
Source: eLife. 2014 Nov 18;3:e02755. doi: 10.7554/eLife.02755 (PMC4235007; doi:10.7554/eLife.02755)
Supplement: Supplementary file 1. — Predicted miR-9 targets screened in miR-9-SpNestin-Cre mice. DOI: http://dx.doi.org/10.7554/eLife.02755.017 [file elife02755s001.docx]

**Supplementary File 1. Predicted miR-9 targets screened in miR-9-Sp^Nestin-Cre^ mice.**

| Gene | Ensembl Gene Id | Prediction databases | | | | Brain HITS-CLIP  Database | Ref. | Upregulated in  miR-9-Sp*^Nestin^* mice? |
| --- | --- | --- | --- | --- | --- | --- | --- | --- |
| Foxp4 | ENSMUSG00000023991 | ● | ● | ● | ● | Yes |  | Yes (*P < 0.05) |
| Creb1 | ENSMUSG00000025958 | ● |  |  |  | No |  | Yes (*P < 0.05) |
| Map1b | ENSMUSG00000052727 | ● |  |  |  | Yes | 1 | Yes (*P < 0.05) |
| Rest | ENSMUSG00000029249 | ● | ● |  |  | No | 2 | Yes (*P < 0.05) |
| Kcna2 | ENSMUSG00000040724 | ● |  |  |  | Yes |  | Yes (*P < 0.05) |
| Pou2f1 | ENSMUSG00000026565 | ● | ● |  | ● | No |  | Yes (*P < 0.05) |
| Nedd4 | ENSMUSG00000032216 | ● |  | ● |  | Yes |  | Yes (*P < 0.05) |
| Gria2 | ENSMUSG00000033981 | ● |  |  |  | Yes |  | Yes (*P < 0.05) |
| Ank2 | ENSMUSG00000032826 | ● |  | ● |  | Yes |  | Yes (*P < 0.05) |
| Kif1b | ENSMUSG00000063077 |  |  |  |  | Yes |  | Yes (*P < 0.05) |
| Mapt | ENSMUSG00000018411 |  |  |  |  | Yes |  | Yes (*P < 0.05) |
| Lmna | ENSMUSG00000028063 | ● | ● | ● | ● | No | 3 | Trend (P=0.072) |
| Bend3 | ENSMUSG00000038214 | ● | ● | ● | ● | No |  | Trend (P=0.086) |
| Gria4 | ENSMUSG00000025892 | ● |  |  |  | Yes |  | NSC |
| Neurod1 | ENSMUSG00000034701 |  |  |  |  | Yes |  | NSC |
| Sirt1 | ENSMUSG00000020063 | ● |  | ● |  | No | 4 | NSC |
| Ankrd11 | ENSMUSG00000035569 |  | ● |  |  | No |  | NSC |
| Pcdh10 | ENSMUSG00000049100 | ● |  |  | ● | No |  | NSC |
| Phr/Mycbp2 | ENSMUSG00000033004 |  |  |  |  | Yes |  | NSC |
| Foxp2 | ENSMUSG00000029563 | ● |  | ● |  | No | 5 | NSC |
| Onecut2 | ENSMUSG00000045991 | ● | ● | ● | ● | No | 6 | NSC |
| Foxg1 | ENSMUSG00000020950 | ● |  | ● |  | No | 7 | NSC |
| Stmn1 | ENSMUSG00000028832 | ● |  |  |  | No | 8 | NSC |
| Apc | ENSMUSG00000005871 |  |  |  |  | Yes |  | NSC |
| Ctnnb1 | ENSMUSG00000006932 | ● |  |  |  | Yes |  | NSC |
| Atrx | ENSMUSG00000031229 |  |  | ● | ● | Yes |  | NSC |

**Supplementary File 1**. Predicted miR-9 targets screened in miR-9-Sp^Nestin-Cre^ mice. The expression levels of predicted miR-9 targets were determined by qPCR in primary hippocampal neurons (DIV3) from miR-9-Sp^Nestin-Cre^ and littermate control embryos. Paired *t* test, n=4, *P < 0.05; NSC: no significant change .Prediction databases: ● miRmap^9^, ● DIANA-microT web server v5.0^10^ ● TargetScanMouse Release 6.2^11^, ● MicroCosm Targets Version 5 (http://www.ebi.ac.uk/enright-srv/microcosm/htdocs /targets/ v5/). Brain HITS-CLIP database^12^.

1. Dajas-Bailador, F. et al. Nat.Neurosci. 15, 697-699 (2012).

2. Packer, A. N., Xing, Y., Harper, S. Q., Jones, L., & Davidson, B. L. J.Neurosci. 28, 14341-14346 (2008).

3. Jung, H. J. et al. Proc.Natl.Acad.Sci.U.S.A 109, E423-E431 (2012).

4. Saunders, L. R. et al. Aging (Albany.NY) 2, 415-431 (2010).

5. Clovis, Y. M., Enard, W., Marinaro, F., Huttner, W. B., & De Pietri, Tonelli D. Development 139, 3332-3342 (2012).

6. Plaisance, V. et al. J.Biol.Chem. 281, 26932-26942 (2006).

7. Shibata, M., Nakao, H., Kiyonari, H., Abe, T., & Aizawa, S. J.Neurosci. 31, 3407-3422 (2011).

8. Song, Y. et al. J.Neurooncol. 115, 381-390 (2013).

9. Vejnar, C. E. & Zdobnov, E. M. Nucleic Acids Res. 40, 11673-11683 (2012).

10. Paraskevopoulou, M. D. et al. Nucleic Acids Res. 41, W169-W173 (2013).

11. Lewis, B. P., Burge, C. B., & Bartel, D. P. Cell 120, 15-20 (2005).

12. Chi, S. W., Zang, J. B., Mele, A., & Darnell, R. B. Nature 460, 479-486 (2009)
